# Implementing, improving and learning: cross-country lessons learned from the African Health Initiative

**DOI:** 10.1186/s12913-017-2655-8

**Published:** 2017-12-21

**Authors:** Lisa R Hirschhorn, Lola Adedokun, Abdul Ghaffar

**Affiliations:** 1 Medical Social Sciences, Feinberg School of Medicine, Northwestern University, 625 N Michigan Ave, 14-013, Chicago, IL 60611 USA; 2 Division of Global Health Equity, Brigham and Women’s Hospital, Boston, MA USA; 3 University of Global Health Equity, Kigali, Rwanda; 4 Doris Duke Charitable Foundation, New York, NY USA; 5 Alliance for Health Policy and Systems Research, World Health Organization, Geneva, Switzerland

Effective health systems strengthening interventions that are able to improve primary health care and generate replicable knowledge to accelerate change are widely recognized as critical to achieving quality universal health coverage. The Ebola outbreak further highlighted the need for effective and resilient primary health care systems that are able to deliver quality services at the local and subnational levels [1]. In parallel, there has been growing recognition that even when effective interventions are discovered, there is a large gap between knowing what works and knowing how to make it work in the real world and in diverse settings [2, 3]. There has also been a recognition of the central role of high quality and people-centered primary health care delivery in determining whether countries have been able to meet the health-related Millennium Development Goals. Ensuring quality primary health care delivery continues to play a central role in efforts to achieve the effective Universal Health Care coverage needed to reach health-related Sustainable Development Goals.

To generate and disseminate actionable knowledge on strengthening primary health care systems, different methodologies are being tested to capture lessons learned from the implementation of complex interventions. These study designs reach beyond the classic randomized control trial and include realist evaluations, adaptive practical trials, applying qualitative methods in combination with quantitative measurement, and case studies and frameworks to better understand how and why complex interventions focused on health systems strengthening did or did not work and where context-specific adaptation may be needed [4, 5]. Implementation research, a discipline designed to study the implementation of evidence-based interventions and the role of contextual factors in determining success while identifying where adaption is needed, can contribute to the production of this evidence needed for policy makers and implementers to accelerate the work to improve health systems and outcomes [6, 7]. This type of program evaluation and research often focuses not on intervention outcomes, but the process and outcomes needed to understand implementation pathways [8, 9].

In 2009, the Doris Duke Charitable Foundation’s African Health Initiative (AHI) funded five partnerships between US-based academic institutions and the public sector in Tanzania, Ghana, Zambia, Mozambique and Rwanda to design, implement and study multifaceted interventions, specific to their country context, to improve primary care. The Initiative also required the integration of implementation research as a means for embedding learning in the service delivery process [10, 11]. The goals of the AHI-funded projects, known as the Population Health Implementation and Training (PHIT) Partnerships, were to 1) improve population health; 2) use embedded implementation research to contribute to the growing knowledge on how to implement health systems strengthening interventions and the role of the local context in how interventions are designed, implemented and adapted, and; 3) build local capacity for implementation research. The funding model included supporting multi-year efforts designed by each country that embedded research into the health system and prioritized a focus on building local research and leadership capacity. This approach of embedding research and building local leaders was designed to ensure that the questions relevant to the implementers and community were being prioritized, that there was local ownership of the results which would be needed to facilitate further adoption and spread, and that a resource was created within each country for ongoing innovation and evidence-based implementation [12].

In 2013, the Partnership teams co-authored a journal supplement that described their individual intervention designs as well as cross-cutting components including improving data and service delivery quality and strengthening health information systems, as well as a common evaluation framework and shared indicators built around the World Health Organization’s six building blocks for health systems strengthening [13–15]. A number of the projects have presented data around outcomes and impacts related to their specific intervention, with analysis ongoing for others. In addition to providing key lessons that are useful for informing the replication and scale of their interventions, project outcomes in Ghana, Mozambique, Rwanda and Tanzania informed national policy design and implementation. Furthermore, project leadership from the Ghana and Mozambique projects have been promoted from regional/provincial leadership roles to national positions, enabling them to directly translate lessons at the national level. Despite differences in the country project contexts and intervention designs, there emerged a number of cross-cutting components, including mentoring to improve clinical service quality and systems [16], improving data quality [17], data utilization for quality improvement [18], neonatal mortality reduction efforts [19], and research capacity building [20]. We recognized an opportunity to harvest the implementation strategies of each project to extract common lessons learned as well as key differences in the design and implementation across multiple countries all working to improve primary care systems and outcomes in the sub-Saharan region. Across the countries, there was agreement to retrospectively evaluate the differences and commonalities within these shared components, borrowing methods from implementation research (frameworks), multiple case studies, and supplementing existing individual country data with qualitative inquiry of key informants and document review.

The papers in this supplement describe the implementation approaches and implementation outcomes in these cross-cutting areas which are critical to accelerate health systems strengthening, including improving data quality and use, improving the quality of care delivered through better supervision, and addressing neonatal mortality, which has persisted as a major cause of lives lost even as under-5 mortality has fallen. Hedt-Gauthier and colleagues [20] report on the cross-cutting lessons and challenges in research capacity building, which was a core priority across the programs. In addition, Sherr et al. describe some of the measurement challenges in these complex interventions [21] and Cyamatare and colleagues report on the cross-cutting intervention and implementation designs and contextual factors associated with the successes and challenges of the projects [22]. This supplement is designed to complement the existing and planned intervention outcome and impact publications of the PHIT projects by providing a wealth of information on the critical lessons learned about how these shared components were implemented across multiple intervention designs and settings, the reported effectiveness of the interventions, and lessons learned.

The supplement concludes with a commentary by the former Director General of the Ghana Health Service on the importance of the approach of embedding implementation research to accelerate knowledge translation from pilots and other research studies into practice. He also highlights the importance of the research findings from the cross-country analyses, which identify core elements of different components of the health systems strengthening work, where adaption is needed to reflect local contexts, and plans for scaling up efforts to improve routine data quality, mentoring, and neonatal mortality.

The results described in the supplement will be valuable to inform policy makers, managers and future implementation researchers on which interventions to choose to strengthen primary health care, how to adapt the components and pathways to implement in their context, and how to effectively evaluate the implementation process and outcomes. It has become evident that these types of analyses created through embedded implementation research are critical for results of health systems strengthening interventions to inform policy and practice [23]. Capturing and disseminating this knowledge is urgently needed to accelerate progress in strengthening effective people-centered integrated primary health care as a critical component of Universal Health Care and necessary to achieve the health-related Sustainable Development Goals.

## Abbreviations

AHI: African Health Initiative
PHIT: Population Health Implementation and Training partnerships
